# Identification of a Novel Substrate-Derived Spermine Oxidase Inhibitor

**DOI:** 10.32607/actanaturae.10992

**Published:** 2020

**Authors:** T. T. Dunston, M. A. Khomutov, S. B. Gabelli, T. M. Stewart, J. R. Foley, S. N. Kochetkov, A. R. Khomutov, R. A. Casero Jr.

**Affiliations:** Sidney Kimmel Comprehensive Cancer Center, The Johns Hopkins University School of Medicine, Baltimore, MD 21287 USA; Engelhardt Institute of Molecular Biology, Russian Academy of Sciences, Moscow, 119991 Russia; Department of Medicine, The Johns Hopkins University School of Medicine, Baltimore, MD 21205, USA; Department of Oncology, The Johns Hopkins University School of Medicine, Baltimore, MD 21287, USA

**Keywords:** spermine oxidase, inhibitors, MDL72527, spermine analogues, 2,11-Met2-Spm

## Abstract

Homeostasis of the biogenic polyamines spermine (Spm) and spermidine (Spd), present in μM-mM concentrations in all eukaryotic cells, is precisely regulated by coordinated activities of the enzymes of polyamine synthesis, degradation, and transport, in order to sustain normal cell growth and viability. Spermine oxidase (SMOX) is the key and most recently discovered enzyme of polyamine metabolism that plays an essential role in regulating polyamine homeostasis by catalyzing the back-conversion of Spm to Spd. The development of many types of epithelial cancer is associated with inflammation, and disease-related inflammatory stimuli induce SMOX. MDL72527 is widely used *in vitro *and *in vivo *as an irreversible inhibitor of SMOX, but it is also potent towards *N*1-acetylpolyamine oxidase. Although SMOX has high substrate specificity, Spm analogues have not been systematically studied as enzyme inhibitors. Here we demonstrate that 1,12-diamino-2,11-bis(methylidene)-4,9-diazadodecane (2,11-Met2-Spm) has, under standard assay conditions, an IC_50_ value of 169 μM towards SMOX and is an interesting instrument and lead compound for studying polyamine catabolism.

## INTRODUCTION

The biogenic polyamines spermine (Spm) and spermidine (Spd), and their diamine precursor putrescine (Put), are organic polycations present in all eukaryotic cells in μM-mM concentrations that *a priori *determine the diversity of their functions, many of which are vitally important [1, 2]. Polyamine intracellular levels are strictly controlled by precise regulation of the activity, biosynthesis and degradation of key enzymes of their metabolism. Polyamines are tightly involved in these regulatory processes, and the cell spends considerable energy to maintain polyamine homeostasis [3]. Disturbances of polyamine metabolism and homeostasis are associated with many diseases [1-6], but they may be most essential to cancer cells, which can have elevated requirements for polyamines. Compounds capable of specifically decreasing the polyamine pool have potential as anticancer drugs [5] and for chemoprevention [6].

**Fig. 1:**
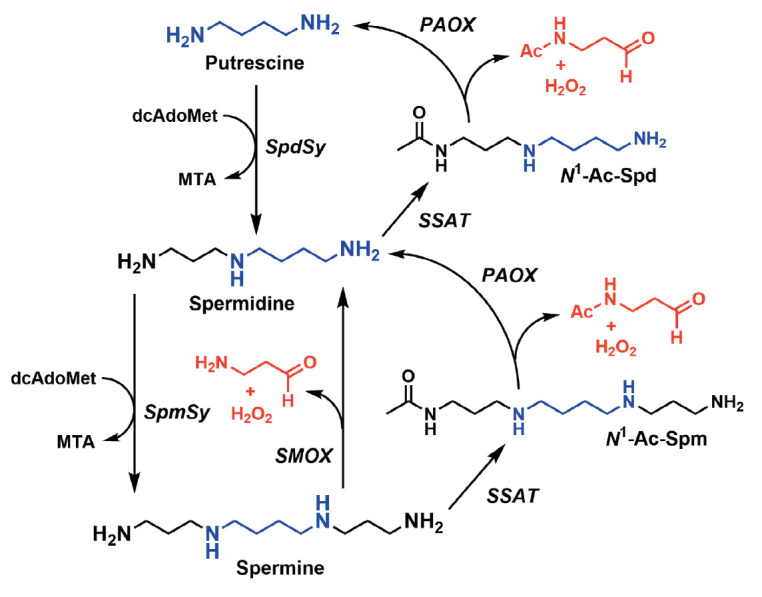
Polyamine interconversions. dcAdoMet – decarboxylated *S*-adenosylmethionine; MTA – 5’-deoxy-5’- methylthioadenosine; PAOX – *N*1-acetylpolyamine oxidase; SMOX – spermine oxidase; SpdSy – spermidine synthase; SpmSy – spermine synthase; SSAT – spermidine/spermine- *N*1-acetyltransferase

FAD-dependent spermine oxidase (SMOX, Fig. 1) converts Spm to Spd with the formation of hydrogen peroxide, a source of ROS, and 3-aminopropanal, which can spontaneously form highly toxic acrolein (Fig. 1). SMOX has been demonstrated to contribute to cancer, including prostate, colon and gastric cancer induced by infection and inflammation [7, 8, 9]. In gastric cancer, Helicobacter pylori infection induces SMOX in gastric epithelial cells that results in the generation of hydrogen peroxide and acrolein-producing 3-aminopropanal; these lead to DNA damage and apoptosis [10]. Inhibition of SMOX with the N1-acetylpolyamine oxidase (PAOX, Fig. 1) irreversible inhibitor MDL72527 {N1,N4-(bis(2,3-butadienyl)-1,4-butanediamine)} [11], which has an IC_50_ value of 90 μM towards SMOX, reduces these effects [8, 9]. However, in some cases it is necessary to discriminate the individual impact of SMOX and PAOX in an integral biological effect or development of the disease and MDL72527, which has been successfully and widely used for decades and inhibits both enzymes. Specific, effective and irreversible inhibitors of SMOX are lacking, partly because the X-ray structure of the enzyme is not available. The analysis of structure/activity relationships of polyamine analogues for PAOX and SMOX has indicated that both enzymes recognize two positively charged amino groups and have hydrophobic pocket(s) located close to the substrate binding site [12]. Therefore, a number of N-substituted diamines were investigated as potential inhibitors of SMOX. However, the problem of specific inhibition of each enzyme has still not been completely solved.

C9-4 (*N*1-nonyl-1,4-diaminobutane) is a Put derivative having an IC_50_ value of 2.6 μM towards PAOX and an IC_50_ value of 88 μM towards SMOX. This compound reduced the volume of brain infarction in a mouse model more effectively than MDL72527 [13]. The *nor*-Spd derivative SI-4650 (*N*-(3-{[3-(dimethylamino) propyl]amino}propyl)-8-quinolinecarboxamide) has an IC_50_ value of 380 μM towards SMOX and an IC_50_ value of 35 μM towards PAOX. SI-4650 inhibited cell growth, induced apoptosis, and promoted autophagy, making it a compound of interest for cancer treatment [12]. Recently, among a family of *N*-substituted 3,5-diamino-1,2,4-triazoles, an efficient and specific inhibitor of SMOX, *N*5-(2-([1,1′-biphenyl]-4-yloxy) benzyl)-1*H*-1,2,4-triazole-3,5-diamine, was identified as having an IC_50_ value of 25 μM (the compound had an IC_50_ value of >200 μM towards PAOX); this compound efficiently inhibited SMOX in cell culture [14]. Currently, this is the one compound that is significantly more effective towards SMOX than PAOX. Moreover, this *N*-substituted 3,5-diamino-1,2,4-triazole is 3.5-fold more potent against SMOX *in vitro *if compared with MDL72527 and is a promising tool to study the effects of specific SMOX inhibition on polyamine metabolism [14].

**Fig. 2:**
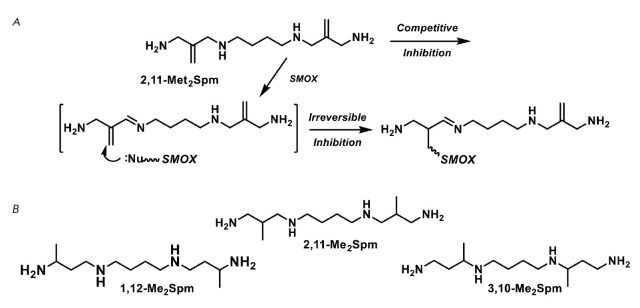
(*A*) Possible mechanism of SMOX inhibition with 2,11-Met2-Spm. (*B*) Structures of *bis*-methylated Spm analogues: 1,12-Me2Spm, 2,11-Me2Spm and 3,10-Me2Spm

Properly designed Spm derivatives/analogues have never been widely studied as specific inhibitors of SMOX. However, taking into consideration that Spm is a substrate of SMOX and not a substrate of PAOX, one may expect that Spm derivatives may be a useful source of specific SMOX inhibitors. In the present paper, we started such investigations using 2,11-Met2-Spm (*Fig. 2A*) for the inhibition of SMOX.

## EXPERIMENTAL

**Materials**

1,12-Diamino-2,11-bis(methylidene)-4,9-diazadodecane tetrahydrochloride (2,11-Met2-Spm) was synthesized essentially as described in [15] starting from 2-chloromethyl-3-chloropropene-1 (Aldrich), which was reacted with potassium phthalimide to give 1-phthalimido-2-methylidene-3-chloropropane, which was used to alkylate bis-*N*1,*N*4-2-nitrophenylsulfonyl- 1,4-diaminobutane. Subsequent removal of protecting groups resulted in 2,11-Met2-Spm in a good overall yield.

**Protein expression and purification**

The bacterial expression vector pET15b carrying the gene coding for the human SMOX protein was used to transform and express SMOX in *E. coli *BL21(DE3) competent cells using Luria Broth (LB) media supplemented with carbenicillin (100 μg/mL), 20 mg/L riboflavin and induced with 0.1 mM IPTG overnight at 18°C. The cells were lysed in a buffer containing 50 mM Na_2_HPO_4_/NaH_2_PO_4_ (pH 8.0), 150 mM NaCl, 10 mM imidazole, 10% glycerol, and 1% Triton X-100. Flavin adenine dinucleotide (FAD) was added at 250 μM with protease inhibitor (1 mM phenylmethylsulfonyl fluoride) and 7 μL β-mercaptoethanol per 10 mL lysis buffer. The lysate was centrifuged at 12,000 rpm for 30 min at 4°C, and the supernatant was applied to a Ni-NTA column. The column was pre-equilibrated with lysis buffer, and the protein was eluted in a gradient in buffer containing 50 mM Na_2_HPO_4_/NaH_2_PO_4_ (pH 8.0), 150 mM NaCl, and imidazole ranging from 50 to 250 mM. To remove the polyhistidine tag, the protein was subjected to thrombin cleavage (25 U) and dialyzed with 10K MWCO snakeskin into buffer containing 100 mM Tris-HCl (pH 7.5) and 50 mM NaCl (with BME) overnight at 4°C. The resulting protein solution was then subjected to Source15Q anion exchange to remove impurities.

**SMOX activity assay and enzyme inhibition studies**

SMOX activity was measured using a chemiluminescent enzyme-based assay detecting the formation of H_2_O_2_ in the presence of Spm as the substrate, as described earlier [16]. To measure the activity of 2,11-Met2-Spm against SMOX, the enzyme (300 ng) in 0.083 M glycine buffer (pH 8.0) and the inhibitor (0–250 μM) were added to the luminol-HRP master mix and incubated at 37°C for 2 min. Spm was then added to the reaction mixture at a final concentration of 250 μM, vortexed for 3 s, and chemiluminescence was integrated over 40 s. Data were averaged and normalized to the blank reaction (no inhibitor) as % SMOX activity. Inactivated SMOX served as a negative control and was accounted for in the calculations.

## RESULTS AND DISCUSSION

**Design of a SMOX inhibitor of Spm origin**

There is a set of different strategies to design suicide inhibitors of the enzymes of amino acid metabolism. One strategy consists in using a substrate/product analogue with a properly positioned activated double bond(s); for example, the allene group in MDL72527, which obeys irreversible inhibition [10]. An activated double bond may be generated at one of the steps of the substrate-like transformation of the inhibitor, like in the case of pyridoxal-5’-phosphate (PLP)-dependent ornithine decarboxylase and its suicide inhibitor DFMO [17]. The subsequent addition of a nucleophile to the activated double bond results in irreversible inhibition, which is developed in time. A double bond may already exist in the structure of the amino acid analogue and become activated as a result of the interaction with the coenzyme, similar to the mechanisms involved with the interaction between α-vinylic amino acids and PLP-dependent enzymes [18]. Here, these considerations were transformed into 1,12-diamino-2,11-bis(methylidene)-4,9-diazadodecane tetrahydrochloride (2,11-Met2-Spm) having a double bond in the beta position to the splitting *C-N *bond (*Fig. 2A*). The methylidene group may be activated as a result of the substrate-like transformation of 2,11-Met2-Spm, leading to the formation of the intermediate Schiff base (*Fig. 2A*). The possibility of substrate-like transformations of 2,11-Met2-Spm is evidenced by the known dependence of the substrate properties of *bis*-methylated Spm analogues in the SMOX reaction on the position of the methyl groups in the analogue structure. The ability of racemic 1,12-Me2Spm, 2,11-Me2Spm and 3,10-Me2Spm (*Fig. 2B*) to serve as substrates for SMOX decreased as the methyl group was positioned closer to the secondary (N4) amino group, and for 3,10-Me2Spm, kinetic parameters were impossible to determine [19]. This is likely because the methyl group at the third position of the Spm backbone may restrict the proton splitting at the C3 carbon atom and influence the formation of the Shiff base, a key intermediate of the SMOX reaction.

**Enzyme inhibition studies**

**Fig. 3:**
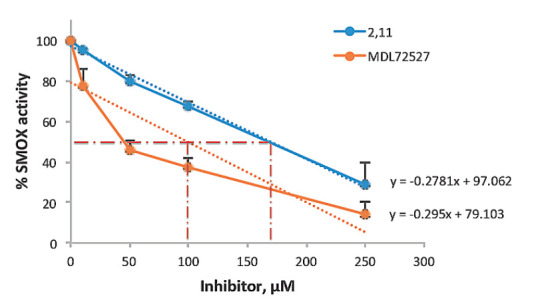
Inhibition of SMOX with 2,11-Met2-Spm (blue line) and MDL72527 (yellow line) as a positive control. Conditions: HRP-luminol (1 ng) in glycine buffer pH 8.0, enzyme and inhibitor (0–250 μM) were incubated at 37°C for 2 min. Spm was then added at a final concentration of 250 μM, and luminescence was integrated for 40 s. 2,11-Met2-Spm and MDL72527 have IC_50_ values of 169 and 100 μM, respectively. Unlike 2,11-Met2-Spm, the inhibition of purified SMOX by MDL72527 does not conform well to a linear transformation but it correlates well with the published IC_50_ value of 90 μM [14]. The R-squared values for 2,11-Met2-Spm an MDl72527 are 0.992 and 0.7821, respectively. Data were collected from three independent experiments with standard deviations (SD)

The experiments on the inhibition of SMOX with 2,11-Met2-Spm were performed under standard assay conditions, preincubating the enzyme with the inhibitor for 2 min and starting the reaction with the addition of Spm: with 250 μM of 2,11-Met2-Spm added, the enzyme was inhibited by 72% (*Fig. 3*). If the inhibition is competitive, the affinity of 2,11-Met2-Spm towards SMOX must be greater than that of Spm (Spm concentration in the substrate mixture was also 250 μM, i.e. 14 Km). High affinity of 2,11-Met2-Spm for SMOX seems unlikely due to the high substrate specificity of the enzyme. Among twenty-nine closely related Spm analogues of tetra- and pentaamine nature, the best substrate was pentaamine 3433 (1,16-diamino-4,8,13-triazahexadecane), with Km of 1.3 μM, i.e. 14 times better than Spm; among the rest, only pentaamine 3434 (1,17-diamino-4,9,13-triazaheptadecane) was as efficient as Spm [20]. However, if the inhibition of SMOX is irreversible, the affinity of the inhibitor towards the enzyme at the reversible stage may be poor, being consistent with the results observed when SMOX was preincubated with 2,11-Met2-Spm at 100 μM and the enzyme activity was inhibited only by 33% (*Fig. 3*). It is currently unclear how quickly inhibition develops in time and the 2 min preincubation time, typical for MDL72527, may be too short for 2,11-Met2-Spm and SMOX because of the steric effect of the methylidene group in the β-position to the splitting *C-N *bond.

The activity of 2,11-Met2-Spm towards SMOX (IC_50_ = 169 μM) was worse than that reported for MDL72527 (IC_50_ = 90 μM [14]), which is an irreversible PAOX inhibitor of a Put nature with reactive allene substituents. As a Spm derivative, it is likely that 2,11-Met2-Spm will be less inhibitory of PAOX (natural substrates are *N*1-Ac-Spd and less effective *N*1-Ac-Spm) compared with SMOX. This is likely based on the comparison of the activity of the structurally similar *rac*-2,11-Me2Spm (*Fig. 2B*) towards SMOX and PAOX. *Rac*-2,11-Me2Spm was a comparatively poor substrate of SMOX, having a Vmax of 124 pmol/min/μg protein and a Km of 121 μM, while the activity of PAOX was inhibited for 60% only at the 500 μM concentration, when a fixed 50 μM concentration of the substrate *N*1-Ac-Spd was used in the PAOX assay [19].

Our results clearly show that it is possible to design a Spm analogue that inhibits the FAD-dependent SMOX, a key enzyme of polyamine catabolism. 2,11-Met2-Spm has an IC_50_ value of 166 μM towards SMOX. Although the precise mechanism of the inhibition, the specificity of 2,11-Met2-Spm action, and the activity in cell culture are under investigation, the development of a selective inhibitor remains critical, not only as an experimental tool, but also as a potential therapeutic agent as SMOX is known to play a critical role in the development of multiple diseases, including cancer [5, 7, 8, 10].

## Abbreviations

Spm: spermine
Spd: spermidine
SMOX: spermine oxidase
PAOX: N1-acetylpolyamine oxidase
MDL72527: {N1,N4-(bis(2,3-butadienyl)-1,4-butanediamine)}
2,11-Met2-Spm: 1,12-diamino-2,11-bis(methylidene)-4,9-diazadodecane
2,11-Me2Spm: 1,12-diamino-2,11-dimethyl-4,9-diazadodecane
